# How Well Do Our Adsorbents Actually Perform?—The Case of Dimethoate Removal Using Viscose Fiber-Derived Carbons

**DOI:** 10.3390/ijerph20054553

**Published:** 2023-03-03

**Authors:** Vladan Anićijević, Tamara Tasić, Vedran Milanković, Stefan Breitenbach, Christoph Unterweger, Christian Fürst, Danica Bajuk-Bogdanović, Igor A. Pašti, Tamara Lazarević-Pašti

**Affiliations:** 1 Military Technical Institute (VTI), Ratka Resanovića 1, 11000 Belgrade, Serbia; 2 VINČA Institute of Nuclear Sciences—National Institute of the Republic of Serbia, University of Belgrade, Mike Petrovica Alasa 12-14, 11000 Belgrade, Serbia; 3 Wood K Plus—Kompetenzzentrum Holz GmbH, Altenberger Strasse 69, 4040 Linz, Austria; 4 Institute of Chemical Technology of Inorganic Materials (TIM), Johannes Kepler University Linz, Altenberger Strasse 69, 4040 Linz, Austria; 5 Faculty of Physical Chemistry, University of Belgrade, Studentski Trg 12-16, 11158 Belgrade, Serbia

**Keywords:** activated carbons, adsorption, organophosphate, adsorption capacity, pollutant uptake

## Abstract

Growing pollution is making it necessary to find new strategies and materials for the removal of undesired compounds from the environment. Adsorption is still one of the simplest and most efficient routes for the remediation of air, soil, and water. However, the choice of adsorbent for a given application ultimately depends on its performance assessment results. Here, we show that the uptake of and capacity for dimethoate adsorption by different viscose-derived (activated) carbons strongly depend on the adsorbent dose applied in the adsorption measurements. The specific surface areas of the investigated materials varied across a wide range from 264 m^2^ g^−1^ to 2833 m^2^ g^−1^. For a dimethoate concentration of 5 × 10^−4^ mol L^−1^ and a high adsorbent dose of 10 mg mL^−1^, the adsorption capacities were all below 15 mg g^−1^. In the case of high-surface-area activated carbons, the uptakes were almost 100% under identical conditions. However, when the adsorbent dose was reduced to 0.01 mg mL^−1^, uptake was significantly reduced, but adsorption capacities as high as 1280 mg g^−1^ were obtained. Further, adsorption capacities were linked to adsorbents’ physical and chemical properties (specific surface area, pore size distribution, chemical composition), and thermodynamic parameters for the adsorption process were evaluated. Based on the Gibbs free energy of the adsorption process, it can be suggested that physisorption was operative for all studied adsorbents. Finally, we suggest that a proper comparison of different adsorbents requires standardization of the protocols used to evaluate pollutant uptakes and adsorption capacities.

## 1. Introduction

Environmental pollution is one of the biggest issues in modern society as it affects many of its different aspects, but the most significant effect is on human health [1,2,3]. Excessive production and use of pesticides cause increases in their levels in wastewater [4]. The pest control industry generates wastewater containing highly toxic pollutants, such as pesticides and solvents used in formulations [5]. In addition, washing tools used for pesticide spraying and rinsing contaminated fruits and vegetables lead to the fortification of wastewater with pesticides.

Surface water and groundwater are becoming highly polluted by industrial, agricultural, and urban wastewater [4]. Pesticide-containing wastewater requires excessive treatment before it can be mixed with other water bodies. Depending on the duration of persistence of certain pesticides, they can remain in the environment for a long time or be transformed into more toxic forms [6]. Organophosphates are the most widely used pesticides today. Due to their ability to inhibit acetylcholinesterase, organophosphate pesticides (OPs) have a detrimental impact on the human central nervous [7] and respiratory systems [8]. In addition to their acute toxicity, organophosphates have a range of undesirable implications for human health, including cancer development [9] and depression [10,11].

Toxic contaminants have to be removed from the environment, and the remediation strategies depend on the nature of the pollutant [12]. Environment remediation strategies involve adsorption [13,14], filtration [15], oxidation [16,17,18], and other chemical and physical treatments [19,20,21], as well as their combinations, such as the combination of adsorption-assisted photochemical degradation [22].

Nevertheless, adsorption is probably the simplest and one of the most effective ways to remediate air, soil, and water [23,24,25]. As general guidance for developing novel adsorbents, the requirements for a high specific surface and open pore structure can be mentioned [26,27]. However, surface chemistry also plays an important role in determining how a pollutant will interact with an adsorbent [28]. Due to their many desirable properties, such as their high surface area, tunable pore structure, adjustable surface chemistry, and generally low price, carbon-based materials, especially nanostructured ones, have found diverse applications as adsorbents for different pollutants [29]. These include zero-dimensional forms (quantum dots) [30], one-dimensional nanotubular forms [31,32], two-dimensional graphene-based materials [33,34,35,36,37], and other forms of carbons, such as biomass-derived carbons [38,39]. One of the key issues in studies on different adsorbents is the evaluation of the adsorbent performance. Different materials are usually compared in terms of pollutant uptake or adsorption capacity. The latter is a parameter that can be derived from different forms of adsorption isotherms or generally calculated for given experimental conditions with a pre-defined amount of adsorbent and pollutant [40]. Adsorption capacity is given in mol or units of mass (mg or g) of pollutant adsorbed per unit mass of adsorbent (usually in g). Thus, the larger the adsorption capacity is, the better the adsorbent performance. However, adsorption capacity is a thermodynamic quantity determined under equilibrium conditions. Thus, it might not properly represent materials’ behavior under realistic conditions.

Viscose fibers have been successfully used as a precursor for activated carbons before. It was shown that the yield and porosity of the viscose and porous carbon fibers could be improved through optimization of the carbonization process in the design of the experiment [41]. The most critical factor for the yield is the heating rate. To increase the specific surface area to the range of commercial activated carbons (2000–3000 m^2^ g^−1^), a chemical activation agent and a physical activation step using water vapor or CO_2_ should be applied. The results obtained in this study indicated that viscose-based precursors are suitable for producing activated carbons with sufficient specific surface areas at high yields. Furthermore, significant yield and performance improvements were noticed when diammonium hydrogen phosphate was used as an impregnating agent [42]. Moreover, different viscose-based activated carbons were applied as adsorbents for OP removal from water [38,39].

In this paper, we show that adsorption capacity is greatly influenced by the experimental setup used to evaluate adsorption performance. To a certain extent, this is also linked to the necessity for the proper choice of standard state for adsorption, as discussed in [43]. In addition, the adsorption capacity is necessary to calculate the adsorption equilibrium constant, which is further used to derive the thermodynamic functions of the adsorption process. Thus, the choice of the standard state and the determination of the adsorption capacity should be uniform if one intends to compare different adsorbents.

Here, we focus on removing organophosphate pesticide dimethoate (2-dimethoxy phosphino thioyl sulfanyl-N-methylacetamide) from aqueous solutions using viscose-derived carbons, some of which were activated after the carbonization process. We chose dimethoate because it is a widely used pesticide soluble in water and represents a significant environmental threat. While it is moderately toxic for mammals, it has been proven to be highly toxic for bees and other pollinators [44]. For adsorbent synthesis, different impregnation steps before the carbonization process and post-carbonization activation were used to introduce significant variation in the specific surfaces, pore volumes, and chemical compositions of the studied adsorbents. However, the morphology was unaffected by the carbon production process. We show that changing the adsorbent dose in the adsorption experiments could result in record-breaking high adsorption capacities (up to 1280 mg g^−1^), while the thermodynamic properties associated with adsorption also depended on the experimental conditions. Adsorption performance was linked with the physical and chemical properties of the studied carbon materials, and we discuss how adsorbent dose affects material performance in realistic remediation cases.

## 2. Materials and Methods

### 2.1. Material Synthesis

All the samples were prepared from viscose fibers (1.7 dtex, 38 mm). All samples were dried for 24 h at 90 °C, and most of them were subjected to an impregnation step before the carbonization process. Impregnation was undertaken using diammonium hydrogen phosphate (DAHP) or ammonium sulfate (AS) in deionized water for 15 min (see Table 1 for details). After impregnation, fibers were spin-dried for 15 min and then stored in a drying cabinet at 90 °C for 24 h. Carbonization was undertaken in a chamber furnace (HTK8, Carbolite Gero GmbH, Neuhausen, Germany) under a nitrogen atmosphere. The heating rates and carbonization temperatures are provided in Table 1. After reaching the final temperature, the samples were kept isothermal for 20 min. Finally, the activation step was performed under different conditions, as described in Table 1. Activation was undertaken in an RSR-B 120/500/11 rotary kiln (Nabertherm GmbH, Lilienthal, Germany) using CO_2_ or H_2_O. The produced carbons were ground in a mortar mill (RM 200, Retsch GmbH, Haan, Germany) and used as-synthesized without additional washing. The sample notations and descriptions are provided in Table 1. ACF is used for activated carbon fibers without impregnation and with carbonization at 850 °C and activation using CO_2_ at 870 °C. CF is used for carbonized fibers without additional activation. Further variations in the standard parameters are given as suffixes in the sample notation.

### 2.2. Materials Characterization

The morphology of the samples was investigated using a PhenomProX scanning electron microscope (Thermo Fisher Scientific, Waltham, MA, USA). The same instrument was used to analyze the chemical composition with energy-dispersive X-ray spectroscopy (EDX).

The specific surface area and textural properties of the obtained ACFs were analyzed via N_2_ isothermal adsorption (−196.15 °C) with a gas sorption system (Autosorb iQ, Quantachrome Instruments, Boynton Beach, FL, USA). The samples were de-gassed for at least 2 h at 200 °C before the analysis. The specific surface area and derived pore size distribution (PSD), along with the cumulative pore volume and mean pore size, were calculated using the Brunauer–Emmett–Teller (BET) method and non-local density functional theory (NLDFT), respectively.

The Raman spectra of the samples were recorded on a DXR Raman microscope (Thermo Fisher Scientific, Waltham, MA, USA). The samples were excited with a diode laser (emission line: 532 nm) with 2 mW of power focused on a 2.1 μm spot on the surface of the sample. The spectrum was obtained as an average of three measurements from different spots on each sample (10 exposures of 10 s each per place).

### 2.3. Adsorption Performance

#### 2.3.1. Adsorption under Equilibrium Conditions

The investigated adsorbents were dispersed in double-distilled water, and the desired amount of dimethoate stock solution (Pestanal, Sigma Aldrich, Søborg, Denmark, https://www.sigmaaldrich.com/RS/en/product/sial/45449, accessed on 26 Ferbruary 2023) was added to provide the targeted adsorbent dose and dimethoate. Then, the vessel containing the adsorbent + dimethoate mixture was placed on a laboratory shaker (Orbital Shaker-Incubator ES-20, Grant Instruments, Royston, UK) and left for 24 h at 25 °C to ensure equilibrium was reached. After equilibration, the mixture was centrifuged for 10 min at 14,500 rpm, and the supernatant was filtered through the nylon filter membrane. The concentration of dimethoate after adsorption (C_eq_) was determined using UPLC analysis (Section 2.3.3). Control experiments were performed identically without carbon adsorbents and confirmed that there was no dimethoate degradation within the timeframes of the described experiments. From the described batch adsorption measurements, we calculated the uptake for the investigated dimethoate with all the studied adsorbents by varying the adsorbent dose and with a fixed dimethoate concentration (*C*_0_ = 5 × 10^−4^ M). Equilibrium uptake (UPT_e_) was calculated as follows:

(1) $${\mathrm{UPT}}_{e}\;(\%)=100\times \frac{C_{0}-C_{e}}{C_{0}}$$

where *C_e_* is the equilibrium concentration of dimethoate determined using UPLC. Equilibrium adsorption capacity (*q_e_*) was calculated as follows:

(2) $$q_{e}=\frac{m_{\mathrm{OP},e}(\mathrm{mg})}{m_{C}(g)}$$

where *m*_OP,e_ and *m*_C_ stand for the mass of adsorbed dimethoate and the mass of carbon adsorbent, respectively.

#### 2.3.2. Filtration Experiments—Adsorption under Dynamic Conditions

Commercial nylon membrane filters were modified to include the layer of adsorbent to analyze dimethoate adsorption during the filtration process, as described in [45]. Briefly, a total amount of 10 mg of each sample was dispersed in 1.5 cm^3^ of deionized water and injected into the commercial filter (KX Syringe Filter, Kinesis, pore size: 220 nm, Cole Parmer, St. Neots, UK). Then, excess water was removed from the carbon-modified filter using compressed air. After that, dimethoate solution (*C*_0_ = 5 × 10^−4^ M) was injected through the modified filter at a rate of 1 mL min^−1^. The total amount of dimethoate was adjusted to match the amount used in the batch adsorption measurements to enable a direct performance comparison. After filtration, the filtrate was subjected to UPLC analysis to determine the concentration of dimethoate. Control experiments were performed using non-modified filters. Furthermore, by comparing pesticide concentrations before and after the filtration through the non-modified filter, we confirmed that the removal of pesticides from the filtrate was not due to the nylon membrane. The efficiency of a modified filter in OP removal was also quantified as the pesticide uptake, denoted UPT_f_:

(3) $${\mathrm{UPT}}_{f}\;(\%)=100\times \frac{C_{0}-C_{f}}{C_{0}}$$

where *C*_f_ is the dimethoate concentration in the filtrate. The adsorption capacity for filtration (*q*_f_) was evaluated as:

(4) $$q_{e}=\frac{m_{\mathrm{OP},f}(\mathrm{mg})}{m_{C}(g)}$$

where *m*_OP,f_ stands for the mass of adsorbed dimethoate in the filtration experiment. All the adsorption measurements were undertaken in triplicate.

#### 2.3.3. Determination of Dimethoate Concentration

A Waters ACQUITY Ultra Performance Liquid Chromatography (UPLC) system coupled with a tunable UV detector controlled with Empower software was used. Chromatographic separations were run on an ACQUITY UPLC™ BEH C_18_ column with dimensions of 1.7 μm and 100 mm × 2.1 mm (Waters, Milford, MA, USA). The analysis of dimethoate was undertaken under isocratic conditions with a mobile phase consisting of 10% acetonitrile and 90% water (*v*/*v*). The eluent flow rate was 0.2 mL min^−1^, and the injection volume was 10 μL. Under the described conditions, the retention time for dimethoate was 2.85 ± 0.05 min. Optical detection of dimethoate was undertaken at 200 nm.

### 2.4. Toxicity Testing

Acetylcholinesterase (AChE) activity was assayed according to a modified Ellman’s procedure [46]. The in vitro experiments were performed by incubating 2.5 IU (commercially purified AChE from electric eel) in treated OP solutions obtained in adsorption experiments (filtered supernatants in batch experiments or filtrates in dynamic adsorption experiments). First, incubation was undertaken in 50 mM PB, pH 8.0, at 37 °C. Then, the enzymatic reaction was initiated by adding acetylcholine-iodide in combination with 5,5′-dithiobis(2-nitrobenzoic acid) (DTNB). DTNB acts as a chromogenic reagent. The reaction was allowed to proceed for 8 min. Then, the reaction was stopped by adding 10% sodium dodecyl sulfate.

Thiocholine, which was the product of the enzymatic reaction between AChE and acetylcholine-iodide, reacted with DTNB and formed yellow-colored 5-thio-2-nitrobenzoate, the absorbance of which was measured as 412 nm. The AChE concentration was kept constant in all experiments as the enzymatic assay had been previously optimized to give an optimal spectrophotometric signal. The toxicity of the treated water samples was quantified via the AChE inhibition as follows:

(5) $$\mathrm{AChE}\;\mathrm{inhibition}(\%)=100\times \frac{A_{0}-A}{A_{0}}$$

where *A*_0_ and *A* stand for the AChE activity in the absence of OP (control) and as measured after exposure to a dimethoate solution, respectively. AChE inhibition measurements were undertaken in triplicate.

## 3. Results and Discussion

### 3.1. Physicochemical Properties

**Figure 1:**
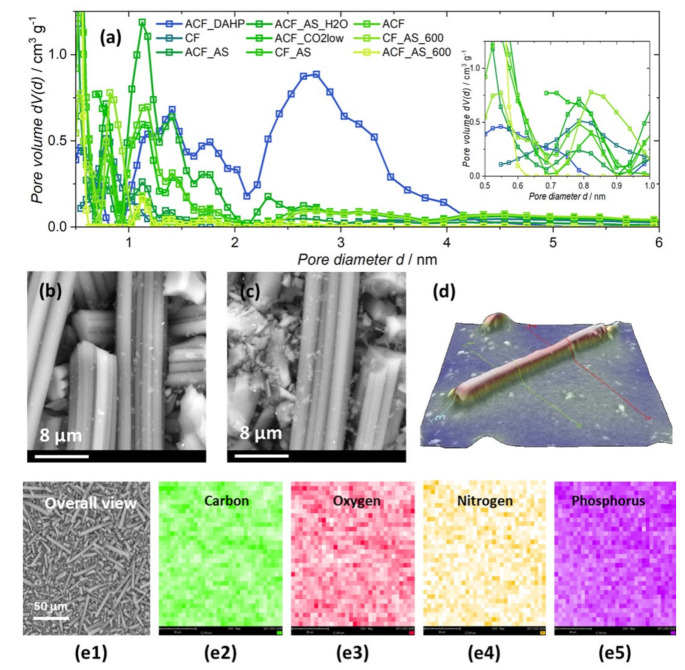
Top row: (**a**) pore size distribution curves for all the studied samples. Middle row from left to right: (**b**) SEM image of ACF, (**c**) SEM image of ACF_AS (field of view was 26.9 μm in both cases), and (**d**) 3D reconstructed single fiber of the sample ACF_AS_H_2_O. Bottom row: (**e1**) low-magnification image of ACF_AS_H_2_O and the corresponding elemental maps obtained with EDX (**e2**–**e5**).

The prepared viscose-derived carbons displayed significant variability in their physical and chemical properties. First of all, their specific surface areas (calculated using the BET equation, *S*_BET_) varied across a wide range from 264 m^2^ g^−1^ to 2833 m^2^ g^−1^ (Table 2). Six out of nine samples were activated in the studied series (Table 1), but only four exhibited *S*_BET_ values above 1000 m^2^ g^−1^. Generally, the total pore volume (*V*_pore_) scaled with the specific surface area rather well (Table 2). However, the situation was somewhat different when considering different ranges (Δ*V*) for the pore size distribution curve (see Table 2 and Figure 1a for pore size distribution (PSD)). As we investigated the adsorption of dimethoate, we chose to consider the pore diameter ranges, which were up to the diameter of one dimethoate molecule (roughly 1 nm), between one and two diameters, and between two and four diameters, in relation to the filling of the pores of the carbon samples during the adsorption process. The correlations were also quite good for the pores below 1 nm and those between 2 and 4 nm, with the sample ACF_DAHP being an outlier in this case. This sample was largely mesoporous, which was in concordance with previous results regarding activated carbon fibers derived from DAHP-impregnated viscose fibers [38,39]. Table 2 assembles the calculated mean pore diameters (*d*_mean_). While the effects of the sample preparation routes can be seen, this property only gives a rough indication of material porosity, and the corresponding PSDs are not unimodal.

All the studied samples retained the overall morphology during the carbonization process (Figure 1b,c). The fibers were found to be slightly below 8 μm in diameter, while they were broken into pieces of different lengths during the gridding step. For example, for the sample ACF_AS_H_2_O, SEM 3D reconstruction of individual fibers showed diameters of 6 ± 2 μm (Figure 1d). Furthermore, all the samples underwent an identical grinding procedure, so there were no reasons to believe that the samples in the studied series had significantly different average fiber lengths. For these reasons, the effects of the samples’ morphology on the adsorption performance can be safely excluded.

The EDX analysis of the studied samples (Table 3) revealed the presence of carbon, oxygen, nitrogen, phosphorus, and, in some cases, small amounts of sodium. Phosphorus was found practically at trace levels, except in the sample ACF_DAHP, the precursor of which was impregnated with DAHP. The same sample had the highest concentration of oxygen, which also suggested the formation of phosphate compounds during carbonization and activation, in agreement with previous observations [38,39,42]. As there was significant overlap between the C and N signals in the EDX results, there might have been some systematic error in the evaluated atomic content of nitrogen, but we did not exclude it from the analysis even in the cases where N was not expected in the samples, as we were interested in overall trends. Nevertheless, higher nitrogen concentrations were found for the samples with precursors impregnated with AS. This was not the case for the ACF_AS_H_2_O sample, which was activated with water steam. Nitrogen-containing compounds were probably washed out during the activation, leading to lower nitrogen content than other samples when the impregnation step with AS was included in the synthesis. For these samples, one might have expected sulfur to be present in higher amounts, but this was not the case. Only traces of sulfur were found in the majority of samples, and the fitting of the EDX spectra for the sulfur line was undertaken with lower reliability than for other elements, except phosphorus. EDX mapping of the samples at low magnification showed a rather uniform distribution of the identified elements (Figure 1(e1–e5)). It should be noted that EDX mapping was undertaken at low magnification (×1.000) to properly average the elemental content in the samples. At higher magnifications, small pieces of debris could be seen on the fibers’ surfaces, but there was no pronounced contrast with the underlying fibers, suggesting that these pieces of debris were the result of sample milling. Higher-magnification EDX maps also supported the relatively uniform distribution of elements, while line scans along individual fibers showed some oscillations in elemental content but no sharp changes (Supplementary Materials Figures S1–S3).

**Figure 2:**
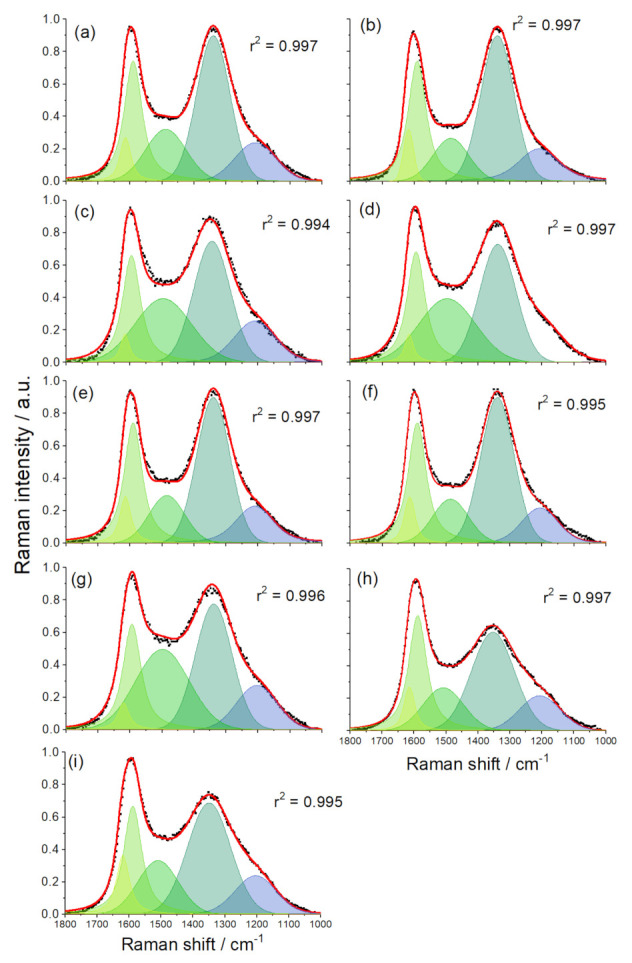
De-convoluted Raman spectra of the studied samples: (**a**) ACF, (**b**) ACF_DAHP, (**c**) ACF_AS, (**d**) CF, (**e**) ACF_CO_2_low, (**f**) ACF_AS_H_2_O, (**g**) CF_AS, (**h**) CF_AS_600, (**i**) ACF_AS_600.

The samples were further probed using Raman spectroscopy (Figure 2). The spectral ranges corresponding to the D and G bands (1000–1800 cm^−1^) were split into five components [47,48]: D (~1340 cm^−1^) and G bands (~1590 cm^−1^), which were fitted using the Gaussian profiles, and an additional three bands denoted D* (~1205 cm^−1^), D″ (~1495 cm^−1^), and D′ (~1615 cm^−1^), for which Voigt profiles were used. All the samples had rather similar *I*_D_/*I*_G_ ratios, and a G band position ranging mainly across the 1590–1600 cm^−1^ window (Table 4), suggesting it was around the border of the stage 1 and stage 2 amorphization trajectory, was presented by nanocrystalline graphite [49], thus indicating a relatively low fraction of sp^3^ domains. Regarding the *I*_D_/*I*_D′_ intensity ratios, four samples had ratios close to 7, which is considered an indication of vacancy-type defects [50]. Another set of samples (ACF_DAHP, ACF_AS, CF, CF_AS) had higher *I*_D_/*I*_D′_ intensity ratios between 9 and 11.9. According to Eckmann et al. [50], an *I*_D_/*I*_D′_ intensity ratio of around 13 indicates an sp^3^-type defect. Thus, these samples could have had a combination of vacancy and sp^3^-type defects. Finally, a low *I*_D_/*I*_D′_ intensity ratio (~3.5) is associated with boundary-type defects, and such a low ratio was only seen for the sample ACF_AS_600, which was both carbonized and activated at the lowest temperature of 600 °C.

### 3.2. Adsorption Performance

First, we evaluated the performance of the studied adsorbents using the uptake (Equations (1) and (3); Table 5). For each batch adsorption experiment, the equilibrium conditions at room temperature (25 °C) were reached by allowing 24 h for equilibration to take place. It was immediately clear that there were essentially two groups of samples. The ones that adsorbed dimethoate to a large extent (high uptake) when the adsorbent dose was high were the samples that were activated. The other group comprised samples with low specific surfaces. The samples with high surface areas performed similarly under equilibrium and filtration conditions, which will be discussed later. In principle, the results are not surprising: as the adsorbent dose decreased, the uptake decreased. The concentration of dimethoate remained the same, while there was less adsorbent. Thus, less dimethoate was removed from the solution when the adsorbent dose was reduced.

**Figure 3:**
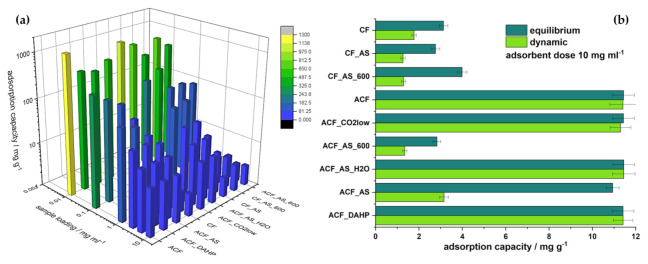
(**a**) Adsorption capacities of studied samples for different adsorbent doses from 10 to 0.01 mg mL^−1^; (**b**) comparison of adsorption capacities under batch and dynamic conditions (filtration) for an adsorbent dose of 10 mg mL^−1^.

To further assess the adsorption performance, we calculated adsorption capacities under equilibrium conditions, analyzing the adsorption process for a constant concentration of dimethoate and varying adsorbent dose. Then, adsorption capacities were evaluated (Figure 3a). For a high adsorbent dose (10 mg mL^−1^), the obtained adsorption capacities were quite modest, ranging up to only ~15 mg g^−1^ for the activated samples with a specific surface above 1000 m^2^ g^−1^. As the adsorbent dose was reduced, the adsorption capacities increased significantly. When the adsorbent doses were in the range of 1 to 0.1 mg mL^−1^, adsorption capacities were in the range typical for pesticide adsorption with carbon materials of between 100 and 200 mg g^−1^. Adsorption capacities were still higher for the activated carbon samples. However, when the adsorbent dose was further decreased, the adsorption capacities further increased, and for the lowest adsorbent dose, they reached rather high values, in some cases above 1000 mg g^−1^. Interestingly, the samples with the highest specific surface areas were not necessarily the best for the lowest adsorbent dose. In fact, it was not the sample ACF_DAHP, which had the highest specific surface, that performed the best but the sample ACF with an adsorption capacity of 1280 mg g^−1^.

This result is very important, as it provides a general context in which the assessment of adsorption performance should be critically addressed. In principle, adsorbents are generally compared in terms of their adsorption capacities, and it is more than clear that a proper comparison requires testing under identical conditions or at least specification of the conditions under which the measurements have been undertaken. Consulting, for example, the relatively old but rather important work by Maliyekkal et al. [34], it is understandable that some astonishing adsorption capacities reported in the literature should be read with care. In the mentioned work, graphene oxide (GO) and reduced graphene oxide (rGO) were used as adsorbents for three different pesticides: endosulfan, chlorpyrifos, and malathion. The former two belong to the same class of organophosphate insecticides as dimethoate. For an adsorbent dose of 3.1 × 10^−3^ mg mL^−1^, the adsorption capacities for chlorpyrifos reached up to 1200 mg g^−1^ with low-concentration pesticide (2 mg L^−1^). For higher adsorbent doses, up to 0.25 mg mL^−1^, the adsorption capacities were very low, in the order of 10 mg g^−1^. Similarly, the importance of the adsorbate concentration for evaluating and comparing adsorption capacities was outlined in [45]. Thus, the adsorbate concentration has to be high enough to ensure saturation of the adsorbent surface under the given experimental conditions—i.e., to reach the plateau of the adsorption isotherm—unless progressive multilayer adsorption takes place. The latter is not common, although it has been reported before [51]. Irrespective of which adsorption isotherm is operative for a given adsorbate–adsorbent combination, the evaluated adsorption capacity will be affected by the adsorbent dose, as there is a dynamic equilibrium under batch adsorption conditions such that, for lower adsorbent doses, the thermodynamic tendency in the adsorption process will be higher. To emphasize the great variation in the adsorption capacities reported in the literature so far, Table 6 compares the performances of some adsorbents for organophosphate removal. As can be seen, adsorption capacities range between 0 and ~1200 mg g^−1^, most of them being in the order of hundreds of mg g^−1^. The real question is whether this comparison gives a real representation of the versatility of the carbon-based adsorbents developed so far or whether it is a consequence of the significantly different approaches and experimental conditions used to assess adsorption capacities.

Before proceeding further, we studied the adsorption process under dynamic conditions; i.e., during the filtering. For this purpose, nylon filters modified with a layer of each studied carbon were used. As there were technical limitations concerning filter modification, it was impossible to use low adsorbent doses. Instead, the adsorbent dose was equivalent to 10 mg mL^−1^, allowing the comparison of dimethoate uptake and adsorption capacities in a straightforward way (Figure 3b). The samples that performed the best in the series under equilibrium conditions were also the best under dynamic conditions. However, only the samples with appreciable *S*_BET_ (>1000 m^2^ g^−1^) had practically the same uptake and adsorption capacities under equilibrium and dynamic adsorption conditions. For example, the sample ACF_AS (*S*_BET_ = 535 m^2^ g^−1^) under equilibrium conditions showed an adsorption capacity close to that of the high-surface-area samples. However, under dynamic conditions, its adsorption capacity was three times lower. Furthermore, all the other samples with low *S*_BET_ showed significantly lower adsorption capacities under dynamic conditions than those determined under equilibrium conditions, which were already quite low, as a large adsorbent dose was used.

**Figure 4:**
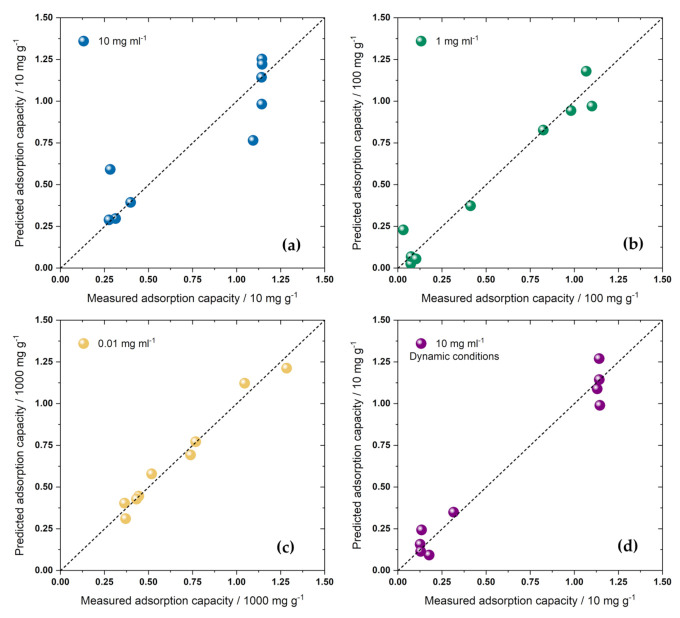
The results of the multiple linear regression model for adsorption capacities determined for adsorbent doses of 10 mg mL^−1^ ((**a**) *R*^2^ = 0.89), 1 mg mL^−1^ ((**b**) *R*^2^ = 0.95), 0.01 mg mL^−1^ ((**c**) *R*^2^ = 0.99), and 10 mg mL^−1^ under dynamic adsorption conditions ((**d**) *R*^2^ = 0.96). Uncertainties for experimental points are omitted for brevity. They did not exceed 8% of the measured values.

Not only did the adsorption capacities greatly depend on the selected adsorbent dose (Figure 3a) but the overall trends also changed with reduction in the adsorbent dose. Dimethoate uptake also showed smaller differences among the samples. The samples with low *S*_BET_ also seemed to perform quite well when the adsorbent dose was low. We calculated adsorbate densities as *q*_e_/*S*_BET_ for different adsorbent doses (Supplementary Materials Figure S4), and no particular correlation with *S*_BET_ was observed except for the lowest dose. In this case, the overall trend suggested that adsorbate density was inversely proportional to *S*_BET_. The correlation coefficient was 0.7 and, for the higher adsorbent doses, below 0.5. This was a clear indication that a single parameter describing material properties is not sufficient to predict adsorption performance. Thus, as in our previous work [38,39,59], we tried to connect the adsorption capacities for different adsorbent doses with the physical and chemical properties of the adsorbents. As the independent variables in the multiple linear regression model, we used *S*_BET_, pore volumes in the three considered pore diameter ranges (Δ*V*; Table 2), and carbon and oxygen content (in at.%; Table 3). In this analysis, we did not consider the effect of mean pore diameter (Table 2). Although this parameter should be considered as important for determining the adsorption process, it is clear that it does not correlate well with adsorption capacities. For example, the ACF_AS_600 sample had the same *d*_mean_ as ACF_AS_H2O, but the latter had an adsorption capacity several times higher. This was likely because the pores of the different sizes were not equally important for the adsorption process, so we used Δ*V* for different pore diameter ranges instead of *d*_mean_. This parameter does not properly reflect material properties if the PSD is not unimodal. The dependent variables were the adsorption capacities for adsorbent doses of 10, 1, and 0.01 mg mL^−1^ for equilibrium conditions and 10 mg mL^−1^ for filtration experiments. To avoid artifacts in the analysis and make all the variables the same order of magnitude, adsorption capacities determined for adsorbent doses of 10, 1, and 0.01 mg mL^−1^ were divided by 10, 100, and 1000, respectively. *S*_BET_ was also divided by 1000, pore volumes were multiplied by 5, and carbon and oxygen content were divided by 100 and 10, respectively. The linearity between the measured adsorption capacities and those that resulted from linear regression was confirmed at the 0.05 level using the ANOVA test. However, it was clear that, for adsorbent doses of 10 mg mL^−1^ (both equilibrium conditions and filtration) and 1 mg mL^−1^, the samples were grouped according to their *S*_BET_ values. Under equilibrium conditions, for an adsorbent dose of 10 mg mL^−1^, the “high surface area group” displayed adsorption capacities around 11 mg g^−1^, and the sample ACF_AS (*S*_BET_ = 535 m^2^ g^−1^) was in this group (Figure 4a). However, for dynamic conditions, this sample was grouped with the low-surface-area samples (Figure 4d). Interestingly, for the adsorbent dose of 1 mg mL^−1^, the sample ACF_AS was between the two groups of samples (Figure 4b).

For the adsorbent dose of 0.01 mg mL^−1^, adsorption capacities were relatively uniformly distributed in the 250–1250 mg g^−1^ range. Multiple linear regression analysis showed a positive correlation of adsorption capacities with *S*_BET_ and carbon content. In the model, a negative correlation was obtained with respect to the pore volumes in specific ranges and for specific levels of oxygen content. However, the overall pore volume was correlated with *S*_BET_ (*R*^2^ > 0.96). Thus, the link between *S*_BET_ and adsorption capacities also included the connection between the pore volume and adsorption capacity. On the other hand, when *S*_BET_ was excluded from the regression analysis, adsorption capacities were positively correlated with Δ*V*_1–2 nm_ and carbon content (*R*^2^ for regression analysis: 0.94). Based on the size of the dimethoate molecule, the pores with *d* > 1 nm were large enough to accommodate it. Thus, a positive correlation between Δ*V*_1–2 nm_ and the adsorption capacity was observed. Carbon and oxygen content were not highly correlated (*R*^2^ > 0.66). Thus, the negative impact of oxygen concentration was a relatively safe conclusion. It might have been that oxygen functional groups on the sample surfaces were highly solvated (hydrated), imposing steric hindrances for dimethoate adsorption and having an overall negative impact on the adsorption capacity in this way.

Although we did not construct adsorption isotherms but analyzed the dependence of the adsorbent dose on the adsorption capacity, a thermodynamic analysis can be provided. Assuming a simple equation corresponding to the adsorption process—DMT_(aq)_ + C→C − DMT_(ads)_, where DMT is the dimethoate molecule in solution (aq) or adsorbed (ads) and C is the adsorption site on the carbon surface—the distribution constant (*K*_dist_) is:

(6) $$K_{\mathrm{dist}}=\frac{q_{e}}{C_{e}}$$

Based on the discussion provided in [43], the distribution constant in Equation (6) is not dimensionless, and the choice of the standard state for the adsorbate in the adsorbed and free state will influence the evaluated thermodynamic parameters. Therefore, we used the standard states proposed in [43]. For the adsorbed layer, we set the standard adsorption capacity as 1 mol kg^−1^(*q*_θ_) and the standard adsorbate concentration as 1 mol L^−1^(*C*_θ_). Then, equilibrium constants (*K*^θ^) were evaluated using these defined standard states (Table 5) as follows:

(7) $$K^{\theta }=\frac{q_{e}}{C_{e}}\times \frac{C_{\theta }}{q_{\theta }}$$

Furthermore, based on the calculated *K*^θ^, we calculated the Gibbs free energy (Δ*G*^θ^) for the adsorption as:

(8) $$\Delta G^{\theta }=-RT\ln K^{\theta }$$

The values obtained for Δ*G*^θ^ (Table 7) suggested an exergonic adsorption process with Δ*G*^θ^ ranging between −26.5 kJ mol^−1^ and −8.6 kJ mol^−1^. The samples were grouped into two clusters for an adsorbent dose of 10 mg g^−1^, with high and low *S*_BET_. However, the grouping diminishes as the adsorbent dose decreased. For the high-surface-area samples, Δ*G*^θ^ became more positive, while for the low-surface-area samples, Δ*G*^θ^ became more negative with decreases in the adsorbent dose. For 0.01 mg mL^−1^ of adsorbent, all Δ*G*^θ^ ranged between −23.4 kJ mol^−1^ and −20.1 kJ mol^−1^. Therefore, the choice of the adsorbent dose also affected the calculated thermodynamic parameters. However, the range of the calculated Δ*G*^θ^ suggested that, in all the cases, relatively weak physisorption was operative, as in the other adsorption studies for carbon adsorbents [43,60].

Finally, to put the applicability of the investigated carbon adsorbents in a proper context, we analyzed the toxicity of purified water samples. Dimethoate inhibited 85% of AChE at the initial concentration (5 × 10^−4^ mol L^−1^). After the treatment of the initial solution with carbons at a dose of 10 mg mL^−1^, for four activated samples, there was no inhibition of AChE, while for an intermediate sample (ACF_AS), the inhibition was rather low (Table 8). Other samples performed quite poorly, and the dimethoate solutions treated with them retained high toxicity. With regard to toxicity reduction, uptake was more relevant than adsorption capacity. Specifically, as the adsorbent dose decreased, the uptake also decreased, so the dimethoate solutions retained a high percentage of the initial toxicity. Thus, although for the adsorbent dose of 0.01 mg mL^−1^, the adsorption capacities reached very high values, they did not mean much in practical terms. For practical applications concerning water purification, it is much more relevant to adjust the adsorbent dose in such a way that the pollutant uptake is maximized. On the other hand, such conditions correspond to low adsorption capacities, at least in our case.

## 4. Conclusions

We have shown that the adsorption performance and evaluated thermodynamic parameters for dimethoate adsorption of viscose-based carbons are highly dependent on the experimental setup. When the adsorbent dose was reduced from 10 to 0.01 mg mL^−1^, the calculated uptake decreased from nearly 100% to just a few percent. On the other hand, the adsorption capacity increased from tens to thousands of mg g^−1^. For the high adsorbent dose of 10 mg mL^−1^, the samples performed similarly under equilibrium conditions and in the filtration experiments. The samples with higher *S*_BET_ (>1000 m^2^ g^−1^) had much higher adsorption capacities than those with low *S*_BET_. On the other hand, for the lowest considered adsorbent dose (0.01 mg mL^−1^), the regression analysis suggested a positive correlation between adsorption capacities and the pore volume for pores with diameters of 1 nm < *d* < 2 nm, as well as carbon content, and a minor influence from *S*_BET_. Oxygen content was found to negatively impact adsorption capacities. The calculated equilibrium constants and Gibbs free energies for dimethoate adsorption showed an *S*_BET_-related grouping for the high adsorbent dose. For the dose of 0.01 mg mL^−1^, all Gibbs free energies ranged between −23.4 kJ mol^−1^ and −20.1 kJ mol^−1^, suggesting dimethoate physisorption for all studied materials. Considering practical applications, the assessment of the treated water samples’ toxicity suggests that high adsorbent doses, corresponding to high dimethoate uptake and low adsorption capacities, should be used in water purification. Thus, materials with *S*_BET_ above 1000 m^2^ g^−1^ should be used for practical applications, as they provide high dimethoate uptake in high doses and significantly reduce the toxicity of contaminated water. Based on the obtained results, we suggest that the protocols for adsorbent performance assessment should be standardized. Specifically, the great diversity in the reported adsorption capacities of different carbon materials is likely due to the different testing protocols rather than tremendous variations in the textural properties and surface chemistry of the different materials.

## Figures and Tables

**Table 1:** The description of the material synthesis and activation conditions.

Sample	Impregnation	Carbonization	Activation
CF	None	850 °C; 10 °C/min	-
CF_AS	12.2 wt.% AS	850 °C; 10 °C/min	-
CF_AS_600	10 wt.% AS	600 °C; 1.0 °C/min	-
ACF	None	850 °C; 5.5 °C/min	870 °C; 165 min; 55 L/h (CO_2_)
ACF_CO_2_low	None	850 °C; 10 °C/min	870 °C; 150 min; 22.5 L/h (CO_2_)
ACF_AS_600	10 wt.% AS	600 °C; 1.0 °C/min	600 °C; 300 min; 94 L/h (CO_2_)
ACF_AS_H_2_O	12.2 wt.% AS solution	850 °C; 10 °C/min	870 °C; 150 min; 0.02 mL/min (H_2_O)
ACF_AS	12.2 wt.% AS	850 °C; 10 °C/min	870 °C; 300 min; 22.5 L/h (CO_2_)
ACF_DAHP	5 wt.% DAHP	850 °C; 5.5 °C/min	870 °C; 165 min; 55 L/h (CO_2_)

**Table 2:** Textural properties of studied carbon materials (*S*_BET_—specific surface area, Δ*V*—pore volumes in specified pore diameter ranges, *V*_pore_—total pore volume, *d*_mean_—average pore diameter).

Sample	*S*_BET_ /m^2^ g^−1^	Δ*V*_0–1 nm_ /cm^3^ g^−1^	Δ*V*_1–2 nm_ /cm^3^ g^−1^	Δ*V*_2–4 nm_ /cm^3^ g^−1^	*V*_pore_ /cm^3^ g^−1^	*d*_mean_ /nm
CF	264	0.085	0.008	0.005	0.116	0.785
CF_AS	380	0.115	0.015	0.018	0.184	0.666
CF_AS_600	430	0.135	0.011	0.016	0.212	0.822
ACF	1568	0.318	0.255	0.020	0.603	1.04
ACF_CO_2_low	1488	0.310	0.225	0.024	0.570	0.718
ACF_AS_600	473	0.162	0.013	0.003	0.186	0.524
ACF_AS_H_2_O	1272	0.295	0.170	0.026	0.518	0.524
ACF_AS	535	0.172	0.028	0.008	0.217	1.49
ACF_DAHP	2833	0.135	0.465	0.950	1.590	2.40

**Table 3:** The elemental composition obtained using EDX analysis (first row: atomic concentration in %, second row: standard deviation over four spots investigated for each sample).

	Atomic Concentration/at.%					
Sample	Carbon	Oxygen	Nitrogen	Sulfur	Phosphorus	Sodium
CF	90.87	6.36	2.73	0.02	0.02	
	2.44	2.40	0.62	0.02	0.01	
CF_AS	82.83	9.76	7.33	0.06	0.03	
	6.44	3.78	3.09	0.06	0.03	
CF_AS_600	86.57	7.17	6.25	0.01	0.01	
	4.52	2.52	2.04	0.01	0.00	
ACF	89.36	8.21	1.62	0.15	0.00	0.67
	1.11	1.12	0.18	0.12	0.00	0.02
ACF_CO_2_low	85.24	11.07	2.29	0.13	0.02	1.25
	2.46	2.27	0.75	0.04	0.01	0.28
ACF_AS_600	86.39	8.05	5.54	0.01	0.01	
	1.37	0.89	0.89	0.00	0.00	
ACF_AS_H_2_O	89.08	8.85	2.03	0.03	0.02	
	3.39	2.90	0.61	0.01	0.01	
ACF_AS	86.76	6.87	6.33	0.04	0.01	
	1.67	1.22	0.66	0.01	0.01	
ACF_DAHP	80.14	14.61	3.49	0.15	1.61	
	1.99	2.49	0.26	0.10	0.24	

**Table 4:** The results for the de-convolution of the Raman spectra of investigated samples.

Function Type	Voigt	Gauss	Gauss	Voigt	Voigt		
Peak Notation	D (D4)	D (D1)	D″ (D3)	G	D′ (D2)		
**Sample**	**Peak Center/Peak Area (a.u.)**					***I*_D_/*I*_G_**	***I*_D_/*I*_D′_**
CF	1209/50	1338/104	1497/87	1594/69	1616/9	1.51	11.6
CF_AS	1203/44	1338/111	1499/105	1594/65	1621/11	1.71	10.1
CF_AS_600	1207/35	1353/103	1509/42	1589/71	1615/16	1.45	6.4
ACF	1208/38	1340/117	1489/51	1591/74	1615/16	1.58	7.3
ACF_CO_2_low	1211/41	1340/117	1485/41	1591/74	1616/18	1.58	6.5
ACF_AS_600	1204/41	1349/116	1509/53	1588/67	1619/27	1.73	4.3
ACF_AS_H_2_O	1207/33	1340/117	1487/38	1591/74	1614/16	1.58	7.3
ACF_AS	1209/45	1344/107	1497/87	1596/66	1616/9	1.62	11.9
ACF_DAHP	1210/39	1340/117	1485/37	1591/74	1618/13	1.58	9.0

**Table 5:** Dimethoate uptake (in %) for the studied samples using different adsorbent doses. Uncertainties in the determined uptake did not exceed 8% of the measured values and were typically under 5%.

	Adsorbent Dose/mg mL^−1^						
Sample	10	5.00	2.50	1.00	0.10	0.01	10 *
CF	27.3	12.6	9.4	6.4	3.6	4.5	15.5
CF_AS	24.1	11.0	9.2	9.6	9.1	3.2	11.0
CF_AS_600	34.8	22.0	9.1	9.0	9.0	6.5	11.3
ACF	99.8	99.7	99.5	93.1	25.5	11.2	99.5
ACF_CO_2_low	99.7	99.4	99.4	85.6	20.5	9.1	98.6
ACF_AS_600	24.7	11.4	7.3	7.7	7.2	3.8	11.7
ACF_AS_H_2_O	99.9	99.8	99.8	96.0	29.4	6.7	99.9
ACF_AS	95.4	97.5	78.2	35.9	9.9	3.2	27.6
ACF_DAHP	99.6	99.1	97.4	71.9	15.6	3.9	99.6

* Dynamic adsorption conditions.

**Table 6:** Comparison of literature data regarding adsorption capacities of organophosphate pesticides.

Pesticide	Adsorbent *	Adsorption Capacity	Reference
Chlorfenvinphos	Graphene-coated silica	4.9 mg g^−1^	[52]
Chlorpyrifos	Cellulose/GO	150 mg g^−1^	[53]
Chlorpyrifos	GO and rGO	Up to 1200 mg g^−1^	[34]
Chlorpyrifos	Biochars	4.32 to 14.8 mg g^−1^	[54]
Chlorpyrifos-methyl		15.0 to 50.5 mg g^−1^	
Chlorpyrifos	Graphene nanoplatelets	140 mg g^−1^	[33]
Chlorpyrifos	Near-single-layer graphene	79 mg g^−1^	
Dimethoate	GO	5.2 mg g^−1^	
Dimethoate	Near-single-layer graphene	37 mg g^−1^	
Dimethoate	Viscose-derived activated carbon fibers	Up to 400 mg g^−1^	[38]
Dimethoate	Activated carbon monoliths	0–46 μg g^−1^	[55]
Diazinon	Walnut shell-modified activated carbon	4.9 to 156 mg g^−1^	[56]
Diazinon	NH_4_Cl-induced activated carbon	Up to 250 mg g^−1^	[57]
Malathion	Granular activated carbon	Up to 900 mg g^−1^	[58]
Dimethoate	Viscose-derived activated carbon fibers	Up to 1280 mg g^−1^	This work

* GO—graphene oxide; rGO—reduced graphene oxide.

**Table 7:** Thermodynamic parameters, equilibrium constants (*K*^θ^), and Gibbs free energy of adsorption (Δ*G*^θ^) determined for adsorbent doses of 10 mg mL^−1^, 1 mg mL^−1^, and 0.01 mg mL^−1^.

	*K*^θ^/Dimensionless			Δ*G*^θ^/kJ mol^−1^		
Sample	10 mg mL^−1^	1 mg mL^−1^	0.01 mg mL^−1^	10 mg mL^−1^	1 mg mL^−1^	0.01 mg mL^−1^
CF	3.76 × 10^1^	6.83 × 10^1^	4.73 × 10^3^	−9.0	−10.5	−21.0
CF_AS	3.18 × 10^1^	7.02 × 10^1^	3.34 × 10^3^	−8.6	−10.5	−20.1
CF_AS_600	7.42 × 10^1^	8.17 × 10^1^	9.22 × 10^3^	−10.7	−10.9	−22.6
ACF	4.34 × 10^4^	1.35 × 10^4^	1.26 × 10^4^	−26.5	−23.6	−23.4
ACF_CO_2_low	3.56 × 10^4^	5.95 × 10^3^	1.00 × 10^4^	−26.0	−21.5	−22.8
ACF_AS_600	5.34 × 10^1^	9.89 × 10^1^	6.89 × 10^3^	−9.9	−11.4	−21.9
ACF_AS_H_2_O	7.13 × 10^4^	2.38 × 10^4^	7.18 × 10^3^	−27.7	−25.0	−22.0
ACF_AS	2.08 × 10^3^	5.61 × 10^2^	3.28 × 10^3^	−18.9	−15.7	−20.1
ACF_DAHP	2.37 × 10^4^	2.56 × 10^3^	4.04 × 10^3^	−25.0	−19.4	−20.6

**Table 8:** AChE inhibition of dimethoate solutions (initial inhibition with 5 × 10^−4^ mol L^−1^ dimethoate solution was 85 ± 2.6%) after the adsorption process with adsorbent doses of 10 mg mL^−1^, 1 mg mL^−1^, and 0.01 mg mL^−1^.

	Adsorbent Dose		
Sample	10 mg mL^−1^	1 mg mL^−1^	0.01 mg mL^−1^
CF	75.0 ± 6.2	81.2 ± 5.8	81.9 ± 5.7
CF_AS	76.2 ± 6.1	81.2 ± 5.8	80.6 ± 5.9
CF_AS_600	68.2 ± 6.2	80.9 ± 5.8	81.5 ± 5.8
ACF	0	10.4 ± 2.1	75.7 ± 6.2
ACF_CO_2_low	0	24.1 ± 1.9	77.3 ± 6.1
ACF_AS_600	72.0 ± 6.2	80.6 ± 5.9	80.6 ± 5.9
ACF_AS_H_2_O	0	5.3 ± 1.8	74.2 ± 6.2
ACF_AS	6.3 ± 1.8	71.5 ± 6.2	80.3 ± 5.9
ACF_DAHP	0	44.3 ± 3.6	78.8 ± 6.0

## Data Availability

Data are available upon reasonable request.

## Supplementary Materials

The following supporting information can be downloaded at: https://www.mdpi.com/article/10.3390/ijerph20054553/s1. Figure S1. EDX maps of sample ACF; Figure S2. EDX maps of sample ACF_AS_H_2_O; Figure S3. EDX line scan along the individual fiber of the samples ACF_AS_600; Figure S4. Correlation between adsorbate density and SBET for different adsorbent doses.

## References

[1] Ghorani-Azam A., Riahi-Zanjani B., Balali-Mood M. (2016). Effects of air pollution on human health and practical measures for prevention in Iran. J. Res. Med. Sci..

[2] Manisalidis I., Stavropoulou E., Stavropoulos A., Bezirtzoglou E. (2020). Environmental and Health Impacts of Air Pollution: A Review. Front. Public Health.

[3] https://www.who.int/news/item/15-11-2019-what-are-health-consequences-of-air-pollution-on-populations.

[4] Saleh I.A., Zouari N., Al-Ghouti M.A. (2020). Removal of pesticides from water and wastewater: Chemical, physical and biological treatment approaches. Environ. Technol. Innov..

[5] Badawy M.I., Ghaly M.Y., Gad-Allah T.A. (2006). Advanced oxidation processes for the removal of organophosphorus pesticides from wastewater. Desalination.

[6] Eichelberger J.W., Lichtenberg J.J. (1971). Persistence of pesticides in river water. Environ. Sci. Technol..

[7] Keifer M.C., Firestone J. (2007). Neurotoxicity of Pesticides. J. Agromed..

[8] Milankovic V., Lazarevic-Pasti T., Dua K. (2021). The Role of the Cholinergic System in Lung Diseases. Targeting Cellular Signalling Pathways in Lung Diseases.

[9] Dich J., Zahm S.H., Hanberg A., Adami H.-O. (1997). Pesticides and cancer. Cancer Causes Control.

[10] Mitić M., Lazarević-Pašti T. (2021). Does the application of acetylcholinesterase inhibitors in the treatment of Alzheimer’s disease lead to depression?. Expert Opin. Drug Metab. Toxicol..

[11] Lazarević-Pašti T., Tasić T., Lazarević-Pašti T. (2022). Organophosphates and Depression. Organophosphates: Detection, Exposure and Occurrence. Volume 1: Impact on Health and the Natural Environment.

[12] Palit S., Hussain C.M. (2020). Chapter 1: Innovation in Environmental Remediation Methods. The Handbook of Environmental Remediation: Classic and Modern Techniques.

[13] Aryee A.A., Mpatani F.M., Han R., Shi X., Qu L. (2021). A review on adsorbents for the remediation of wastewater: Antibacterial and adsorption study. J. Environ. Chem. Eng..

[14] Gusain D., Bux F. (2021). Batch Adsorption Process of Metals and Anions for Remediation of Contaminated Water.

[15] Abdelrasoul A., Doan H., Lohi A., Hironori N. (2013). Fouling in Membrane Filtration and Remediation Methods. Mass Transfer.

[16] O’Shea K.E., Dionysiou D.D. (2012). Advanced Oxidation Processes for Water Treatment. J. Phys. Chem. Lett..

[17] Ameta S.C., Ameta R. (2018). Advanced Oxidation Processes for Wastewater Treatment: Emerging Green Chemical Technology.

[18] Hodges B.C., Cates E.L., Kim J.-H. (2018). Challenges and prospects of advanced oxidation water treatment processes using catalytic nanomaterials. Nat. Nanotechnol..

[19] Li F.B., Li X.Z., Ao C.H., Lee S.C., Hou M.F. (2005). Enhanced photocatalytic degradation of VOCs using Ln3+–TiO_2_ catalysts for indoor air purification. Chemosphere.

[20] Li J., Wang Y., Tian Y., He X., Yang P., Yuan M., Cao Y., Lyu J. (2018). Crystallization of microporous TiO_2_ through photochemical deposition of Pt for photocatalytic degradation of volatile organic compounds. Environ. Sci. Pollut. Res..

[21] Mitrović T., Lazović S., Nastasijević B., Pašti I.A., Vasić V., Lazarević-Pašti T. (2019). Non-thermal plasma needle as an effective tool in dimethoate removal from water. J. Environ. Manag..

[22] Lv J., Zhu L. (2013). Highly efficient indoor air purification using adsorption-enhanced-photocatalysis-based microporous TiO_2_ at short residence time. Environ. Technol..

[23] Wang S., Sun H., Ang H.M., Tadé M.O. (2013). Adsorptive remediation of environmental pollutants using novel graphene-based nanomaterials. Chem. Eng. J..

[24] Mammadova S., Nasibova A., Khalilov R., Mehraliyeva S., Valiyeva M., Gojayev A.S., Zhdanov R.I., Eftekhari A. (2022). Nanomaterials application in air pollution remediation. Eurasian Chem. Commun..

[25] Franco P., Cardea S., Tabernero A., De Marco I. (2021). Porous Aerogels and Adsorption of Pollutants from Water and Air: A Review. Molecules.

[26] Bernal V., Giraldo L., Moreno-Piraján J.C. (2018). Physicochemical Properties of Activated Carbon: Their Effect on the Adsorption of Pharmaceutical Compounds and Adsorbate–Adsorbent Interactions. C.

[27] Hoang N.B., Ngo T.C.Q., Tran T.K.N., Lam V.T. (2022). Comprehensive review on synthesis, physicochemical properties, and application of activated carbon from the Arecaceae plants for enhanced wastewater treatment. Open Chem..

[28] Abegunde S.M., Idowu K.S., Adejuwon O.M., Adeyemi-Adejolu T. (2020). A review on the influence of chemical modification on the performance of adsorbents. Resour. Environ. Sustain..

[29] Sabzehmeidani M.M., Mahnaee S., Ghaedi M., Heidari H., Roy V.A.L. (2021). Carbon based materials: A review of adsorbents for inorganic and organic compounds. Mater. Adv..

[30] Agarwal S., Sadeghi N., Tyagi I., Gupta V.K., Fakhri A. (2016). Adsorption of toxic carbamate pesticide oxamyl from liquid phase by newly synthesized and characterized graphene quantum dots nanomaterials. J. Colloid Interface Sci..

[31] Dehghani M.H., Niasar Z.S., Mehrnia M.R., Shayeghi M., Al-Ghouti M.A., Heibati B., McKay G., Yetilmezsoy K. (2017). Optimizing the removal of organophosphorus pesticide malathion from water using multi-walled carbon nanotubes. Chem. Eng. J..

[32] Peng J., He Y., Zhou C., Su S., Lai B. (2021). The carbon nanotubes-based materials and their applications for organic pollutant removal: A critical review. Chin. Chem. Lett..

[33] Lazarević-Pašti T., Anićijević V., Baljozović M., Anićijević D.V., Gutić S., Vasić V., Skorodumova N.V., Pašti I.A. (2018). The impact of the structure of graphene-based materials on the removal of organophosphorus pesticides from water. Environ. Sci. Nano.

[34] Maliyekkal S.M., Sreeprasad T.S., Krishnan D., Kouser S., Mishra A.K., Waghmare U.V., Pradeep T. (2013). Graphene: A Reusable Substrate for Unprecedented Adsorption of Pesticides. Small.

[35] Kumar S., Parekh S.H. (2020). Linking graphene-based material physicochemical properties with molecular adsorption, structure and cell fate. Commun. Chem..

[36] Paton-Carrero A., Sanchez P., Sánchez-Silva L., Romero A. (2022). Graphene-based materials behaviour for dyes adsorption. Mater. Today Commun..

[37] Verma S., Kim K.-H. (2022). Graphene-based materials for the adsorptive removal of uranium in aqueous solutions. Environ. Int..

[38] Jocić A., Breitenbach S., Pašti I.A., Unterweger C., Fürst C., Lazarević-Pašti T. (2022). Viscose-derived activated carbons as adsorbents for malathion, dimethoate, and chlorpyrifos—Screening, trends, and analysis. Environ. Sci. Pollut. Res..

[39] Jocić A., Breitenbach S., Bajuk-Bogdanović D., Pašti I.A., Unterweger C., Fürst C., Lazarević-Pašti T. (2022). Viscose-Derived Activated Carbons Fibers as Highly Efficient Adsorbents for Dimethoate Removal from Water. Molecules.

[40] Mozaffari Majd M., Kordzadeh-Kermani V., Ghalandari V., Askari A., Sillanpää M. (2022). Adsorption isotherm models: A comprehensive and systematic review (2010–2020). Sci. Total Environ..

[41] Breitenbach S., Unterweger C., Lumetzberger A., Duchoslav J., Stifter D., Hassel A.W., Fürst C. (2021). Viscose-based porous carbon fibers: Improving yield and porosity through optimization of the carbonization process by design of experiment. J. Porous Mater..

[42] Breitenbach S., Lumetzberger A., Hobisch M.A., Unterweger C., Spirk S., Stifter D., Fürst C., Hassel A.W. (2020). Supercapacitor Electrodes from Viscose-Based Activated Carbon Fibers: Significant Yield and Performance Improvement Using Diammonium Hydrogen Phosphate as Impregnating Agent. C.

[43] Chen T., Da T., Ma Y. (2021). Reasonable calculation of the thermodynamic parameters from adsorption equilibrium constant. J. Mol. Liq..

[44] Anićijević V.J., Petković M., Pašti I.A., Lazarević-Pašti T.D. (2022). Decomposition of Dimethoate and Omethoate in Aqueous Solutions—Half-Life, Eco-Neurotoxicity Benchmarking, and Mechanism of Hydrolysis. Water Air Soil Pollut..

[45] Lazarević-Pašti T.D., Pašti I.A., Jokić B., Babić B.M., Vasić V.M. (2016). Heteroatom-doped mesoporous carbons as efficient adsorbents for removal of dimethoate and omethoate from water. RSC Adv..

[46] Ellman G.L., Courtney K.D., Andres V., Featherstone R.M. (1961). A new and rapid colorimetric determination of acetylcholinesterase activity. Biochem. Pharmacol..

[47] Sadezky A., Muckenhuber H., Grothe H., Niessner R., Pöschl U. (2005). Raman microspectroscopy of soot and related carbonaceous materials: Spectral analysis and structural information. Carbon.

[48] Claramunt S., Varea A., Lopez-Diaz D., Velázquez M.M., Cornet A., Cirera A. (2015). The importance of interbands on the interpretation of the Raman spectrum of graphene oxide. J. Phys. Chem. C.

[49] Ferrari A.C., Robertson J. (2000). Interpretation of Raman spectra of disordered and amorphous carbon. Phys. Rev. B.

[50] Eckmann A., Felten A., Mishchenko A., Britnell L., Krupke R., Novoselov K.S., Casiraghi C. (2012). Probing the Nature of Defects in Graphene by Raman Spectroscopy. Nano Lett..

[51] Bondžić A.M., Lazarević Pašti T.D., Pašti I.A., Bondžić B.P., Momčilović M.D., Loosen A., Parac-Vogt T.N. (2022). Synergistic Effect of Sorption and Hydrolysis by NU-1000 Nanostructures for Removal and Detoxification of Chlorpyrifos. ACS Appl. Nano Mater..

[52] Liu X., Zhang H., Ma Y., Wu X., Meng L., Guo Y., Yu G., Liu Y. (2013). Graphene-coated silica as a highly efficient sorbent for residual organophosphorus pesticides in water. J. Mater. Chem. A.

[53] Suo F., Xie G., Zhang J., Li J., Li C., Liu X., Zhang Y., Ma Y., Ji M. (2018). A carbonised sieve-like corn straw cellulose–graphene oxide composite for organophosphorus pesticide removal. RSC Adv..

[54] Zheng H., Zhang Q., Liu G., Luo X., Li F., Zhang Y., Wang Z. (2019). Characteristics and mechanisms of chlorpyrifos and chlorpyrifos-methyl adsorption onto biochars: Influence of deashing and low molecular weight organic acid (LMWOA) aging and co-existence. Sci. Total Environ..

[55] Vukčević M., Kalijadis A., Babić B., Laušević Z., Laušević M. (2013). Influence of different carbon monolith preparation parameters on pesticide adsorption. J. Serb. Chem. Soc..

[56] Bayat M., Alighardashi A., Sadeghasadi A. (2018). Fixed-bed column and batch reactors performance in removal of diazinon pesticide from aqueous solutions by using walnut shell-modified activated carbon. Environ. Technol. Innov..

[57] Moussavi G., Hosseini H., Alahabadi A. (2013). The investigation of diazinon pesticide removal from contaminated water by adsorption onto NH_4_Cl-induced activated carbon. Chem. Eng. J..

[58] Jusoh A., Hartini W., Endut A. (2011). Study on the removal of pesticide in agricultural run off by granular activated carbon. Bioresour. Technol..

[59] Lazarević-Pašti T., Jocić A., Milanković V., Tasić T., Potkonjak N., Breitenbach S., Unterweger C., Fürst C., Pašti I.A. (2022). Kinetics of Dimethoate, Malathion, and Chlorpyrifos Adsorption on Cellulose-derived Activated Carbons–Linking Performance to the Physicochemical Properties. Preprints.

[60] Hamed M.M., Rizk H., Ahmed I. (2018). Adsorption behavior of zirconium and molybdenum from nitric acid medium using low-cost adsorbent. J. Mol. Liq..

