# Supplementary material for: How Well Do Our Adsorbents Actually Perform?—The Case of Dimethoate Removal Using Viscose Fiber-Derived Carbons
Source: Int J Environ Res Public Health. 2023 Mar 3;20(5):4553. doi: 10.3390/ijerph20054553 (PMC10001822; doi:10.3390/ijerph20054553)
Supplement: Supplementary file 1 [file ijerph-20-04553-s001.zip › ijerph-2233976-supplementary.pdf]

Supplementary Information

How well do our adsorbents actually perform? – the case of dimethoate removal using viscose fibers-derived carbons

Vladan Anićijević[1,2], Tamara Tasić[2], Vedran Milanković[2], Stefan Breitenbach[3,4], Christoph Unterweger[3], Christian Fürst[3], Danica Bajuk-Bogdanović[5], Igor A. Pašti[5], and Tamara Lazarević-Pašti[2,*]

[1] Military Technical Institute (VTI), Ratka Resanovića 1, 11000 Belgrade, Serbia
[2] VINČA Institute of Nuclear Sciences—National Institute of the Republic of Serbia, University of Belgrade, Mike Petrovica Alasa 12-14, 11000 Belgrade, Serbia
[3] Wood K Plus—Kompetenzzentrum Holz GmbH, Altenberger Strasse 69, 4040 Linz, Austria
[4] Institute of Chemical Technology of Inorganic Materials (TIM), Johannes Kepler University Linz, Altenberger Strasse 69, 4040 Linz, Austria
[5] Faculty of Physical Chemistry, University of Belgrade, Studentski Trg 12-16, 11158 Belgrade, Serbia
* Correspondence: lazarevictlj@yahoo.com or tamara@vin.bg.ac.rs

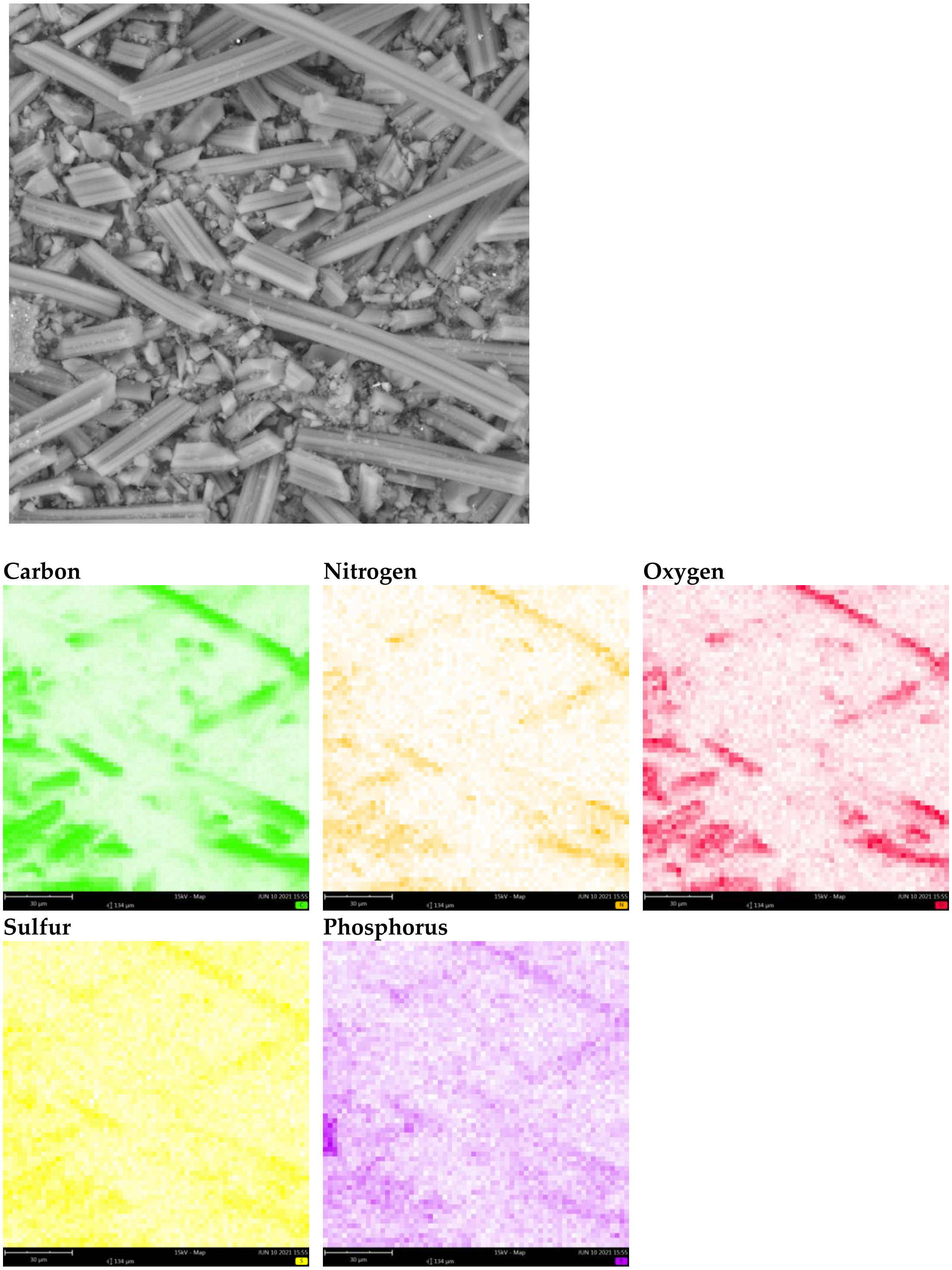

Figure S1. EDX maps of sample ACF

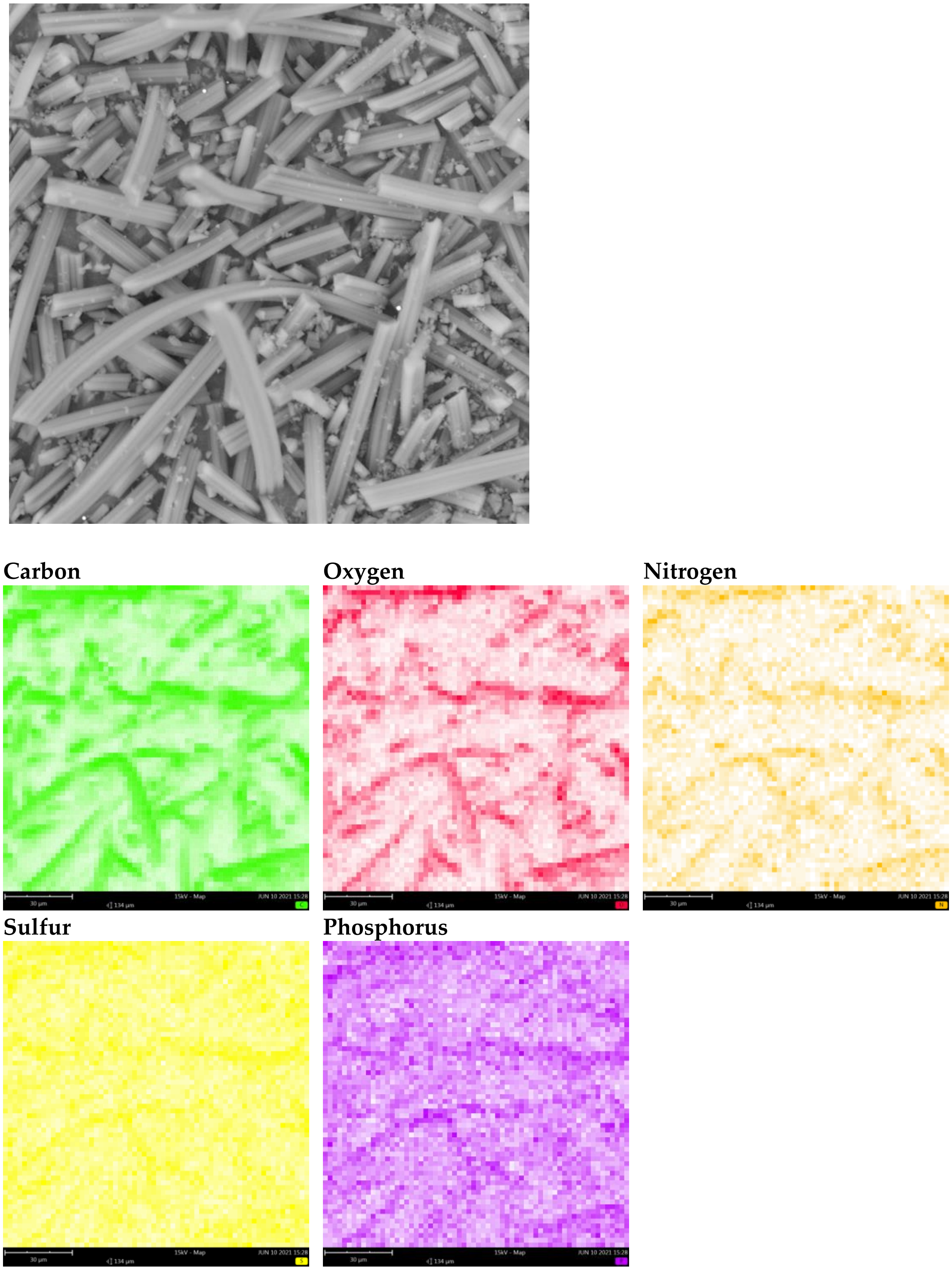

Figure S2. EDX maps of sample ACF_AS_H2O

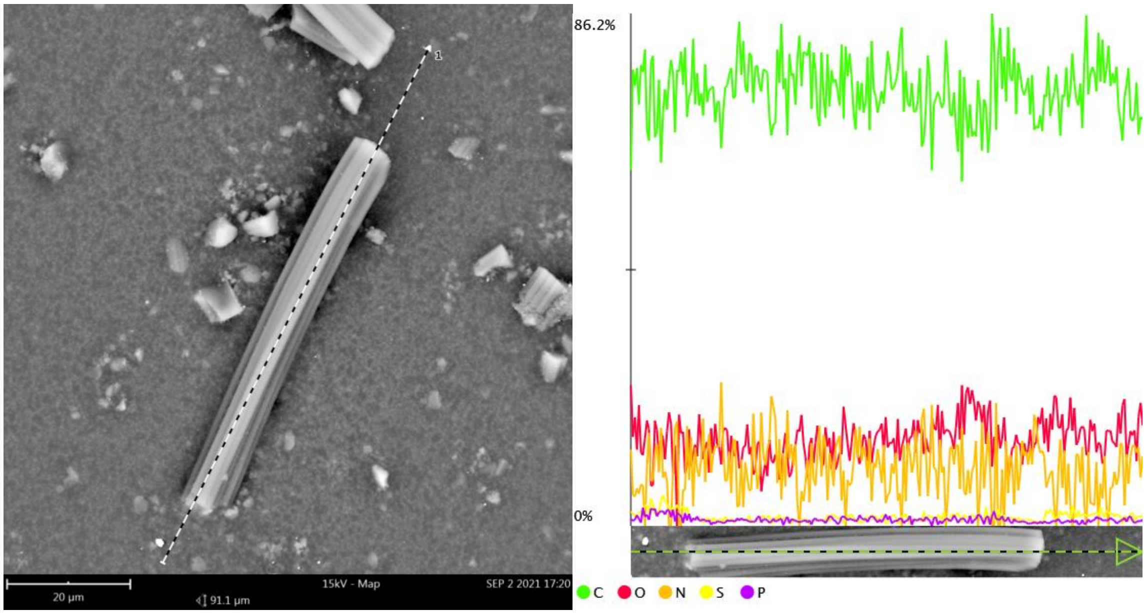

Figure S3. EDX line scan along the individual fiber of the samples ACF_AS_600

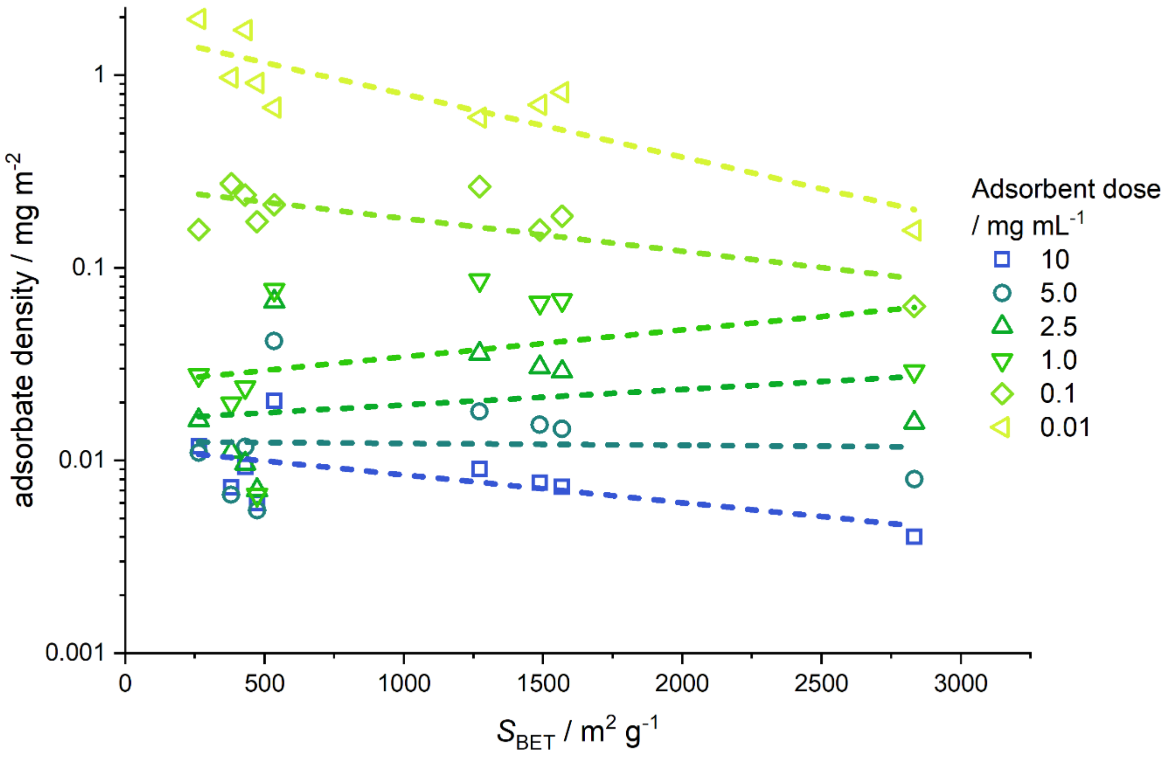

Figure S4. Correlation between adsorbate density and S_{BET} for diferent adsorbent doses.
